# Evaluation of Scintillator Detection Materials for Application within Airborne Environmental Radiation Monitoring

**DOI:** 10.3390/s19183828

**Published:** 2019-09-04

**Authors:** Matthew Lowdon, Peter G. Martin, M.W.J. Hubbard, M.P. Taggart, Dean T. Connor, Yannick Verbelen, P.J. Sellin, Thomas B. Scott

**Affiliations:** 1 Interface Analysis Centre, School of Physics, HH Wills Physics Laboratory, University of Bristol, Tyndall Avenue, Bristol BS8 1TL, UK; 2 Department of Physics, University of Surrey, Guildford, Surrey GU2 7XH, UK

**Keywords:** radiation, NORM, mapping, contamination, scintillators, nuclear, UAV

## Abstract

In response to the Fukushima Daiichi Nuclear Power Plant accident, there has occurred the unabated growth in the number of airborne platforms developed to perform radiation mapping—each utilising various designs of a low-altitude uncrewed aerial vehicle. Alongside the associated advancements in the airborne system transporting the radiation detection payload, from the earliest radiological analyses performed using gas-filled Geiger-Muller tube detectors, modern radiation detection and mapping platforms are now based near-exclusively on solid-state scintillator detectors. With numerous varieties of such light-emitting crystalline materials now in existence, this combined desk and computational modelling study sought to evaluate the best-available detector material compatible with the requirements for low-altitude autonomous radiation detection, localisation and subsequent high spatial-resolution mapping of both naturally occurring and anthropogenically-derived radionuclides. The ideal geometry of such detector materials is also evaluated. While NaI and CsI (both elementally doped) are (and will likely remain) the mainstays of radiation detection, LaBr_3_ scintillation detectors were determined to possess not only a greater sensitivity to incident gamma-ray radiation, but also a far superior spectral (energy) resolution over existing and other potentially deployable detector materials. Combined with their current competitive cost, an array of three such composition cylindrical detectors were determined to provide the best means of detecting and discriminating the various incident gamma-rays.

## 1. Introduction

As a consequence of the graphic and globally significant severe reactor accidents at both the Chernobyl Nuclear Power Plant (ChNPP—1986), and the Fukushima Daiichi Nuclear Power Plant (FDNPP—2011), some of the greatest levels of environmental radioactivity are associated with accidents that occurred at power-generating nuclear sites. Although regarded by the public as the largest contributor to environmental radioactivity, with significant levels of contamination released by both incidents [1], the greatest anthropogenic source and contributor to environmental radioactivity is associated with historic weapons testing [2]. However, unlike the aforementioned reactor releases, this material was distributed globally in contrast to the less spatial-extensive (but higher total activity) plumes that were dispersed into the surrounding regions from both the Chernobyl and Fukushima accidents. Such highly localised and elevated activity anomalies are also associated with localities around the world where reprocessing, fuel generation and defence facilities formerly existed [3].

Despite these significant contributors to environmental radioactivity, the incident radiation typically encountered during routine radiation surveys is a consequence of naturally occurring radioactive materials (NORM); ore and deposits; either concentrated on, or just below, the surface. These include primary and secondary ores containing U, Th and K—in addition to economically-viable accessory elements (e.g., Cu, Sn, and Rare Earth Elements—REEs).

This work evaluates both existing and potentially deployable scintillator-type radiation detection devices for their applicability and performance in monitoring both naturally occurring, as well as anthropogenically-derived radionuclides, distributed within the environment. As a source’s correct identification and provenancing are essential, the performance of the scintillator (both in terms of its efficiency and sensitivity) is crucial in producing discrete peaks within the gamma-ray spectrum at the correct emission energies.

The principles that underpin radiation mapping are to locate and identify the source(s) of radioactivity within the environment or defined area of interest, and to then quantify the intensity. Such objectives can be achieved by various means, using different surveying and data collection methods—which, at the highest level, can be divided into either; (i) ground-based surveys, or, (ii) airborne surveys.

Ground-based radiometric surveying utilises a closely spaced network of measurement points and a semi-portable detection system [4,5]. While this method produces good results for every data point along the survey route, the rate of coverage is low when trying to achieve a good spatial resolution. Likewise, spatial resolution suffers at higher rates of ground coverage. This can result in significant data gaps, which then require interpolation. In some cases, this may lead to hotspots not being identified [4,5]. These surveys are often used to calibrate and validate measurements taken by other radiation survey methods (such as those described in Tyler et al. (1996) [4]); or in situations where absolute spatial accuracy outweighs the need for enhanced spatial coverage.

Ground-based surveys can be undertaken by both humans and vehicles. As such, there exists a large disparity in the rate of spatial coverage. Foot-based surveys represent the lower end of this capability, with typical rates of coverage of approximately 5,000 m^2^hr^−1^ [6]. At the opposing end of this capability are vehicle-hosted surveys, which are able to cover much larger areas, but are confined to the road network [5]. A significant advantage of vehicular-based surveys is the larger detector that can be carried. Larger volume detectors provide a better absolute detection efficiency (and sensitivity) for gamma-rays than smaller volume detectors of the same type, due to the increased photon stopping potential.

Historically, airborne radiation surveys have utilised large crewed aircraft and correspondingly large volume detectors (approximately 33 L and 75–80 kg [7]), and are, therefore, considered the “industry standard” for environmental radiation mapping at a large/catchment-wide scale. The rate of coverage is typically 10^3^ times greater than that achieved using equivalent vehicle-based surveys [5,8], while also yielding more consistent survey patterns—avoiding the aforementioned problems associated with limited data density in some inaccessible areas. While this method has numerous advantages, there do exist drawbacks. Principally, the altitude at which crewed aircraft surveys must operate results in reduced spatial resolution over those performed at ground-level. This is a consequence of the detectors inherent field-of-view (visualised via a “cone of incidence”) whereby, as the distance from the ground increases, so too does the area of ground being sampled. At a nominal survey altitude of 122 m OD, a standard NaI(Tl) detector yields a strip of width (defined as the horizontal distance on the ground from which 75% of the recorded counts will be emanating) of approximately 390 m—produced from a circle of investigation of 195 m radius (× 1.6 the survey altitude) [9]. This resolution could be improved by operating at a lower altitude (but this isn’t always possible or practical) or by introducing considerable measurement overlap and performing subsequent deconvolution to locate areas of interest.

Advancements in uncrewed aerial vehicles (UAVs) have presented new opportunities in low-altitude radiological surveying. The main advantage of which is the improved spatial resolution that these platforms offer—the result of the lower altitude at which they operate [5]. UAVs are also considerably cheaper than larger aircraft and can be operated without the requirement for trained pilots—utilising a large amount of inbuilt autonomy and extensive safety features [5].

Because an increase in the UAVs payload weight dramatically reduces the available flight time, and therefore, the potential area mappable on a single survey flight, the principal challenge for UAVs is the diminished payload capacity that can be carried in contrast to larger manned platforms [5]. Although the larger autonomous helicopter-style systems utilised by Towler et al. (2012) [10] possessed a payload capacity of approximately 20 kg, for the more conventional multi-rotor UAV systems, the maximum payload is only a few kilograms (with the exact value depending upon the individual system). In miniaturising the payload for use on a UAV, the corresponding active detector volume must also be reduced.

One potential mechanism through which to overcome the problems faced by miniaturising detection systems, principally by reducing active detector volume and consequently the detector’s sensitivity, is to explore the wealth of currently available detector materials, or those which could be developed. This variation in detector type is evidenced by the previous works, including; Towler et al. (2012) [10], who utilised a NaI scintillator detector; Sanada and Torii (2015) [11] who employed an array of three LaBr_3_:Ce detectors; and recent works by the University of Bristol [12,13,14,15,16], which have used either CsI(Tl) or a Cadmium Zinc Telluride (CZT) semiconductor detector. Although outside of the payload capacity of a UAV, the work of Šálek, Matolín and Gryc (2018) [17], demonstrated the use of Bismuth Germanate (BGO) detectors in identifying U and Th anomalies.

Consequently, this study simulates different scintillator materials and configurations to establish; (i) the most appropriate radiation detection materials and, (ii) their layout through which to perform radiation mapping when used within the payload for a UAV platform. The ideal detector material and configuration thereof would combine a high energy resolution with a high detection efficiency, providing for good overall system performance, even at reduced detector volumes and system weights.

## 2. Materials and Methods

### 2.1. Candidate Detector Materials

A good energy resolution and detection efficiency are vital when considering smaller volume radiation detection systems. To attain the best energy resolution, the detector material should produce a high optical yield—a quantity which is measured as the number of optical photons emitted per MeV of energy deposited into the crystal volume by the intercepted gamma-ray photon. As this optical generation is a strongly statistical process, the energy resolution of the detector (defined as the full width at half maximum—FWHM) correlates with the intensity of the light yield. The detection efficiency of a scintillator is a function of its ability to attenuate photons and is expressed in terms of the mass attenuation coefficient (µ). As well as the energy of the incident gamma-ray, the mass attenuation coefficient is proportional to both the density and the atomic (Z) number of the material. Hence, detectors composed of Cs (Z = 55) and La (Z = 57) have greater radiation “stopping power” than those of lower densities and Z-numbers, with the detector efficiencies of Gd (Z = 64) containing GAGG detectors being higher still. A summary of several common detector materials is given in Table 1.

The active detection material is not the sole consideration when designing a complete scintillator detector system, whereby the optical coupling of the output of the scintillating crystal to the associated light sensor serves to fundamentally define the inherent output of the system. This light sensor can consist of either the traditional photomultiplier tube (PMT), or the more modern silicon photomultiplier (SiPM). To maximise the optical photon yield, the peak emission wavelength of the scintillator should match the spectral quantum efficiency of the light sensor. Both PMT and SiPM sensors have comparable quantum efficiencies; therefore, scintillator materials emitting between 400 nm and 500 nm (blue-green region) have the best optical match. A comparison of the peak wavelength emission (nm) for a range of scintillator materials, as well as the light yield (photons per MeV), and attenuation at 1.5 MeV (mass attenuation) is detailed in Table 1.

Airborne surveys throughout history have historically employed either one of two scintillator materials; CsI or NaI (both of which exist doped with Tl). The incorporation of dopant at such trace amounts (0.005–0.5% and 0.04–0.05%, respectively [41]) into the crystal structure of the detector materials serves to modify the emission wavelength of the emitted light to match the quantum efficiency of a PMT or SiPM. 

### 2.2. Experimental Conditions

**Figure 1:**
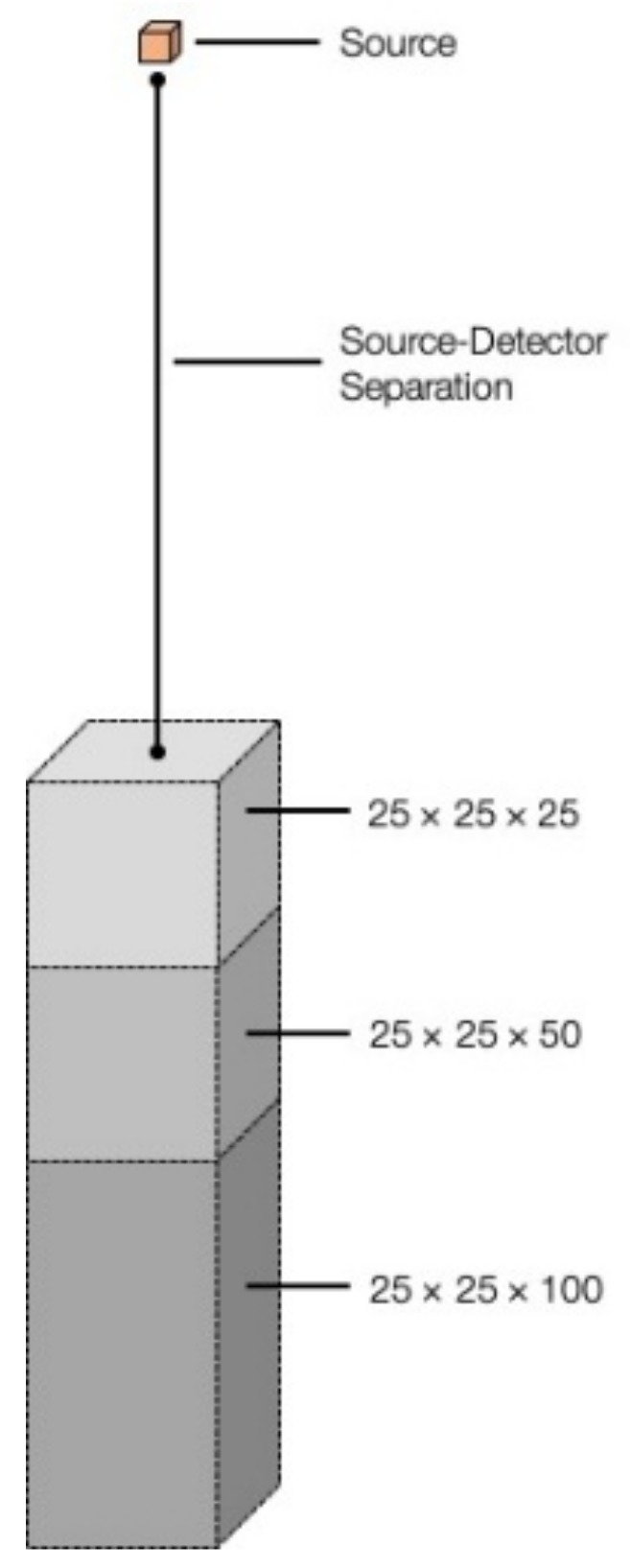
Source-detector geometry used in GEANT4 simulations depicting the varying detector volumes with constant source separation.

A range of simulations were undertaken during this work, each using the GEANT4 software package developed at CERN [42]. The GEANT4 package is a comprehensive toolkit based on the C++ programming language for tracking the transport of particles through various types of user-defined matter. These simulations can be run over a TeV energy range and through an infinite number of different source-detector geometries. A schematic of the source-detector geometry is shown in Figure 1—with the range of differing detector thicknesses held, in each instance, 25 m away from the radioactive point source emitter. The photon emissions from this source of infinitely small dimension were transmitted as a single particle beam directly towards the detector. This geometry served not only to speed up simulation runs, but to also eliminate and influence of gamma-ray divergence (geometric spreading) that normally occurs and would otherwise require a normalisation correction to be applied. The simulation mechanism employed by the GEANT4 package is such that a source activity (intensity) is not defined (e.g., MBq, GBq)—rather the duration of the experiment and number of individual photon (or defined particle) interactions, leading to an evaluation of a sources’ intensity with time. 

Several detector characteristics are explored within the simulation environments. Initially, the effect of varying the volume of the detector was explored, by gradually increasing the thickness of the detector while keeping the cross-sectional area of the face of the detector constant. The simulated detectors were then exposed to pure sources of ^137^Cs, ^232^Th, and ^238^U, and analysed in terms of the resultant spectra. These three emission sources were selected as both ^232^Th, and ^238^U (^208^Tl and ^214^Bi, respectively) are the primary decay peaks associated with geological deposits of uranium-bearing ore minerals, with ^137^Cs the principal gamma-ray emitting radionuclide associated with a nuclear release scenario (e.g., Fukushima and Chernobyl). The influence of the atmosphere was also explored over the same source-detector geometries by simulating both in a vacuum and in standard atmospheric conditions. Finally, the influence of increasing detector volume through the use of a parallel array of separate detectors was assessed. 

## 3. Results and Discussion

### 3.1. Detector Thickness

**Figure 2:**
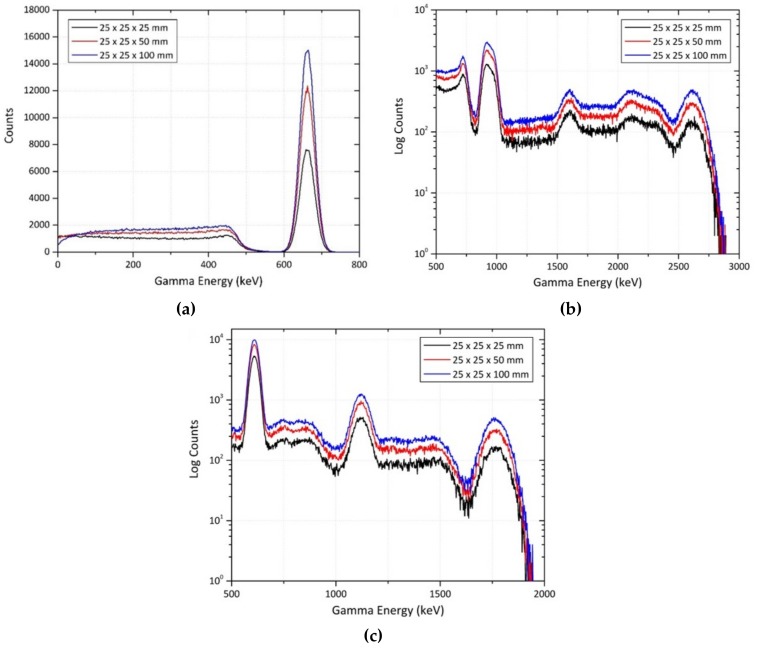
GEANT4 simulated CsI(Na) detector response for varying thicknesses when exposed to; (**a**) ^137^Cs point source; (**b**) ^232^Th point source; (**c**) ^238^U point source.

The GEANT4 simulation results evaluating the response of differing detector thicknesses (all composed of CsI(Na) in this instance) when exposed to point sources of ^137^Cs, ^232^Th and ^238^U, are shown in Figure 2. Looking initially at Figure 2a, for increased detector (volume) profiles, there is a growth observed in the magnitude of the primary 0.662 MeV emission peak, along with an increase in the magnitude of the Compton scattering (at energies less than 0.475 MeV). An approximate doubling of the peak intensity is apparent between the 25 mm and 50 mm thick detectors—a consequence of the increased gamma-ray photon absorption by the greater volume of detector material, resulting in fewer undetected photons escaping from the detector volume having not been fully absorbed. This doubling of the peak intensity, however, is not observed when the thickness of the detector is further increased to 100 mm. A feature, such as this, can be attributed to the maximum CsI(Na) thickness to fully absorb the incident radiation being less than 100 mm—with no further photon detection obtained when a threshold thickness value (< 100 mm) is exceeded. This behaviour can also be seen in Figure 2b,c, where count intensities increase with detector volume.

### 3.2. Air vs. Vacuum Response

**Figure 3:**
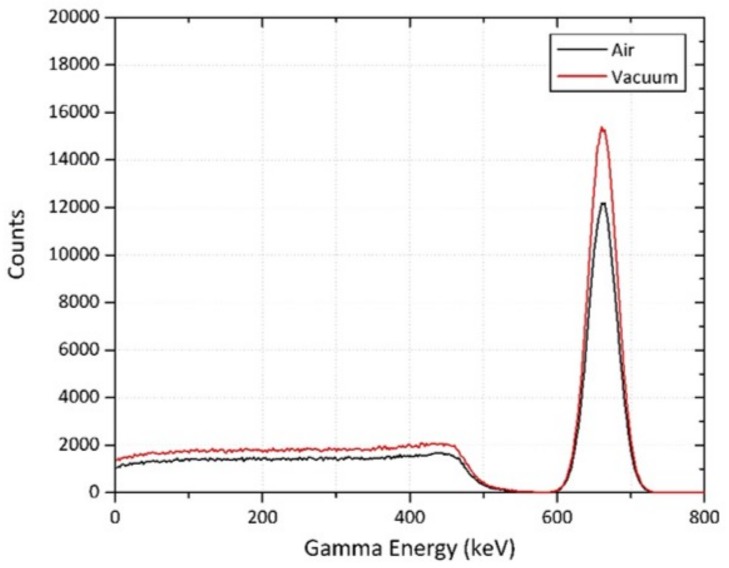
CsI(Na) detector response in “air” and “vacuum” environments.

As shown previously, the influence of the thickness (and therefore volume) of a detector has a quantifiable influence on the proportion of the (emitted) radiation detected. Serving as a point of interest, but also to provide a mechanism through which to validate and quantify the experimental models, further simulations were carried out. The same GEANT4 simulations as described in Section 3.1 for a 25 mm × 25 mm × 50 mm CsI(Na), in the “air” medium, were used in a “vacuum” (air-less) environment. The results of this comparative analysis are shown in Figure 3, for both source-detector environments. From this plot, a 22% increase in the total counts is observed—illustrating the influence of the short distance of air on attenuating a notable component of the incident radiation.

### 3.3. Detector Material

**Figure 4:**
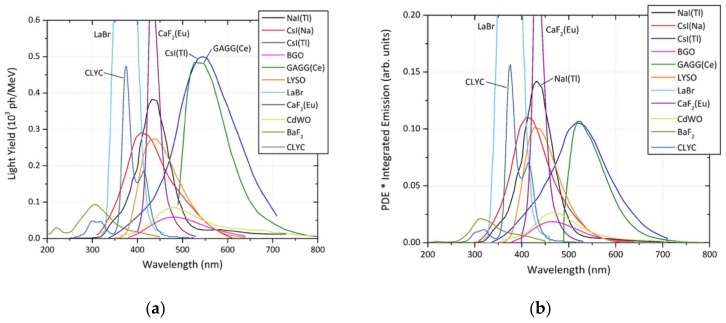
Optical emission characteristics of various scintillation materials; (**a**) absolute photon yield against the wavelength of peak emission; (**b**) effective total optical yield, accounting for the quantum efficiency of the light detector, against the wavelength of maximum emission.

Figure 4 shows the GEANT4 simulation results for the optical emission characteristics of various composition detectors. Highlighted in the plot is the wavelength band over which the PMT/SiPM operates at the greatest (light to electrical) conversion efficiency (400–500 nm). The significant and broad light yield peak of one of the favoured detector types, CsI(Tl), is observed alongside comparable light yield emission characteristics from the GAGG material at the larger wavelengths of 540 nm and 520 nm, respectively. Despite both materials providing a high light yield, the wavelength of this light is not optimally coupled to that of the associated light sensor. However, other detector materials exist that are better matched to the peak emission conversion wavelengths in the range of 375 nm to 465 nm, several of which produce considerable light yields (Table 1). Two of these scintillator detectors are LaBr_3_ and CaF_2_(Eu) (and to a lesser extent, CLYC), which offer a high light yield and subsequently energy resolutions superior compared to the traditional NaI(Tl) and CsI(Tl) (2.6% FWHM for LaBr_3_, rather than the 5.6% FWHM for NaI(Tl)). In contrast, both BGO and BaF_2_ are shown in Figure 4 to provide very low light yields, and therefore, poor energy (spectral) resolution.

To fully evaluate the complete detector system (scintillator crystal plus PMT/SiPM), the light sensor quantum efficiency must also be included. This provides a quantity hereby termed the “effective total optical yield” (of the detector)—the results of this correction are shown in Figure 4b. The graph has been normalised by the spectral quantum efficiency of a SensL J-series SiPM, which has a peak in its quantum efficiency at between 420–470 nm. As shown in Figure 4b, the influence of this coupling serves to reduce the effective optical yield of both CsI(Tl) and GAGG, due to their larger emission wavelengths, and therefore, non-optimal coupling to either PMT or SiPM. Through consideration of the constituents of this plot, four different scintillator materials were chosen to be further investigated as part of this study. These materials are; CsI(Na), CsI(Tl), LaBr-_3_ and LYSO (typically Ce-doped).

Beginning with the GEANT4 simulation results for a single CsI(Na) detector, the gamma-ray spectra plots for point sources of ^137^Cs, ^232^Th and ^238^U, shown in Figure 2, yield two important observations. Firstly, and as discussed further in Section 3.1, that increased detector volumes lead to increased count intensities. Secondly, the plots highlight the poor spectral resolution of CsI(Na), and is particularly well-illustrated by the failure of CsI(Na) to resolve the two ^232^Th emission peaks at 0.911 MeV and 0.968 MeV (^228^Ac daughter peaks), shown in Figure 2b. The result instead is a broad, asymmetrical peak between 0.8 and 1 MeV. At the higher end of the spectrum, the peak at 2.61 MeV (^208^Tl daughter emission) exists across an energy window of 0.196 MeV—between 2.513 MeV and 2.709 MeV. This is mirrored in the gamma spectrum obtained from a ^238^U point source, shown in Figure 2c, whereby each of the three emission peaks at 0.609 MeV, 1.12 MeV and 1.76 MeV (^214^Bi) show widths of 0.08 MeV, 0.111 MeV and 0.150 MeV, respectively.

**Figure 5:**
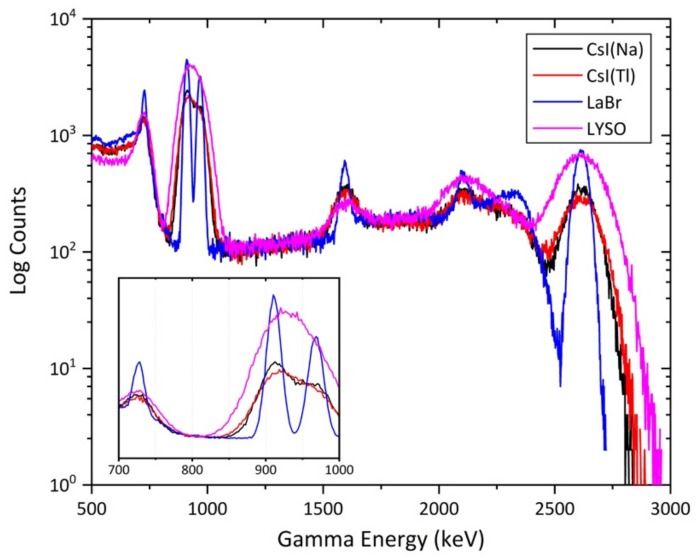
Comparison of the gamma-ray spectra obtained from various detector materials of identical volume when exposed to a ^232^Th point source.

The comparison of the results of the spectral modelling between the CsI(Na) and the other detector materials identified for further investigation, of fixed volume when exposed to a ^232^Th point source, is shown in Figure 5. Apparent from this plot are several key differences between the spectra derived using the different composition detector materials. Despite different dopants being contained within each CsI detector, the simulated output from both is near identical. The marginally elevated counts seen for CsI(Na) over the CsI(Tl) material results from the greater effective optical yield, accounting for the quantum efficiency of the light detector, as shown in Figure 4b. Figure 5 also shows the greater detection efficiency of the LYSO(Ce) composition detector over both CsI-based detectors, evident most clearly in the region between 2.5 and 3.0 MeV of the ^232^Th gamma-ray spectra. Despite the elevated count rates shown by this material, its poor spectral resolution, as shown in Table 1 (8% FWHM at 0.662 MeV), results in similarly broad peaks as for the CsI-based detectors—shown by the single wide peak between 2.5 MeV and 3.0 MeV in addition to the further inability to resolve the two sharp peaks at 0.911 MeV and 0.968 MeV, with the production of only a single, largely symmetrical peak.

In contrast to the CsI(Na), CsI(Tl) and LYSO(Ce) detectors, the LaBr_3_ material is observed to exhibit increased detector efficiency, resulting in greater count rates/peak heights, and substantially enhanced spectral resolution. Both features are apparent in the gamma-ray spectrum, shown in Figure 5. Alongside the greater magnitude peaks, unlike the formerly described materials, the GEANT4 simulated LaBr_3_ spectrum is able to clearly discriminate the two peaks at 0.911 and 0.968 MeV, while substantially reducing the peak width of the 2.61 MeV gamma-ray emission. This due to the excellent energy resolution of 2.6% FWHM at 0.662 MeV characteristic of this material (Table 1).

**Figure 6:**
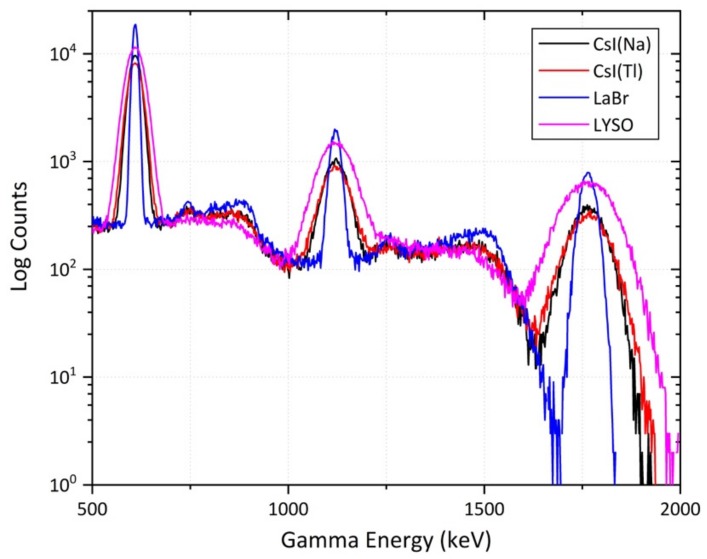
Comparison of the gamma-ray spectra obtained from various detector materials of identical volume when exposed to a ^238^U point source.

Comparable results are further shown in Figure 6, whereby the same four detector materials (each of identical volume) were exposed to a ^238^U point source. Similar to Figure 5, both the CsI-based and the LYSO(Ce) detectors produced spectra with broad, indiscriminate peaks–much wider than those obtained from the same source by the LaBr_3_ detector. The LaBr_3_ detector similarly shows peak counts much greater than for the other detector materials. Despite ^238^U exhibiting a far simpler gamma-ray spectrum than ^232^Th–with only three primary emission peaks at 0.609 MeV, 1.12 MeV and 1.76 MeV the broad nature of the contributing peaks of this, as well as the ^232^Th spectra when analysed using the CsI(Na), CsI(Tl) and LYSO(Ce) detector materials illustrates the inability of each to discriminate closely spaced peaks–especially those associated with mixed (and more environmentally-representative sources). Consequently, a detector composed of LaBr_3_ is, hence, shown to represent one of the best options for environmental surveying of gamma-ray emitting radionuclides based on currently available scintillator materials, when combined with typical PMTs.

#### Material Stability of Lanthanide Halides

Another material of note, though not covered within the scope of this paper, is CeBr_3_ (due to its comparable properties and performance to LaBr_3_). Its reported characteristics are comparable to those of LaBr_3_, with an absolute light yield of 57,000 photons/MeV-66,000 photons/MeV and an energy resolution of 3.8–4% (Table 1). However, this is all without the inherent self-radioactivity in LaBr_3_, which arises as a result of the ^138^La decay to either ^138^Ba or ^138^Ce. These decays produce a 1.436 MeV or 0.789 MeV peak respectively [43], which are useful (an example is in the calibration of gain drift), but increase signal to noise ratio when measuring the 1.461 MeV ^40^K emission and other low energy decays. Furthermore, this provides another factor in the spectral stripping problem, arising from Compton scattering. In CeBr_3_, this inherent radioactivity is almost a magnitude of order less over the range between 0–3 MeV [44].

A further consideration in the choice of scintillator material is its behaviour and chemical stability in a given environment. While LaBr_3_ and CeBr_3_ are arguably the best candidates for UAV applications, their hygroscopic behaviours cannot be ignored. Due to their high deliquescence, LaBr_3_ and CeBr_3_ form LaBr_3_·(H_2_O)_8_ and CeBr_3_·(H_2_O)_7_ respectively at 303 K on exposure to water [45]. This is particularly worrisome for detector purposes, since hydration observed in LaBr_3_ caused rapid degradation in performance and increased susceptibility to cracking [46]. One possible way to mitigate the hygroscopic behaviours of LaBr_3_ and CeBr_3_ is to embed them within a protective, optically matched resin.

### 3.4. Detector Number

The aforementioned high-altitude aerial surveys used to analyse the distribution of environmentally distributed gamma-ray emitting radionuclides have been performed not only at altitudes greater than those of UAV surveys, but also using detectors of larger dimensions (> 30 l [6]) than those modelled in this work. Therefore, to match the high efficiency and resolution of scintillator detectors like LaBr_3_, the increase in sensitivity arising from a greater volume of detector material (through an increased number of 25 mm [diameter] × 100 mm [length] cylindrical CsI(Na) detectors) was further simulated within the same GEANT4 environment. The selection of simulation parameters for this further modelling was the culmination of a number of contributing factors. Such geometries (e.g., lengths, diameters) were selected as they represented currently commercially available detectors (or volumes that could be produced) with weights capable of transportation by the desired UAV platform. The incorporation of 3 smaller volume detectors to produce a large sensor volume rather than a single (larger) volume results from the difficulties in growing such a solid (perfect) volume.

**Figure 7:**
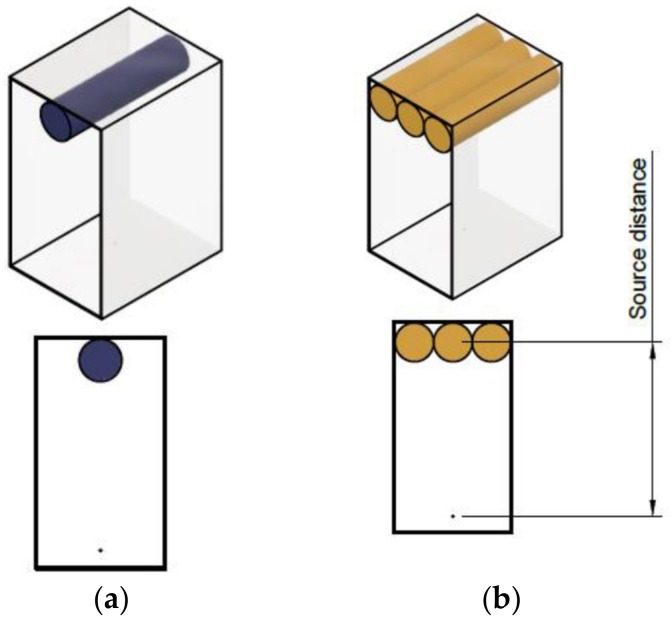
Schematics for; (**a**) single CsI(Na) detector setup; (**b**) triple CsI(Na) detector setup.

**Figure 8:**
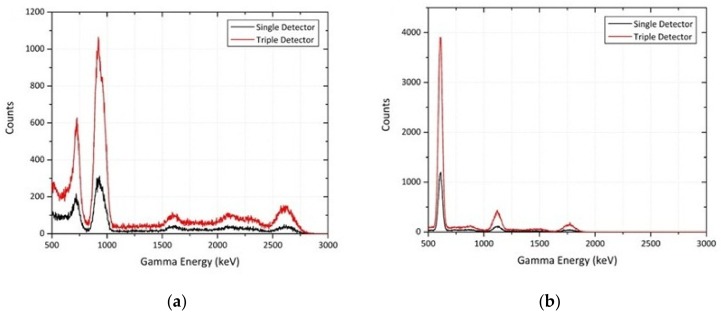
Gamma spectra obtained using a single and triple CsI(Na) detector setup for: (**a**) ^232^Th point source; (**b**) ^238^U point source.

The results of these simulations for source compositions of ^232^Th and ^238^U, detected using either one or an array of three parallel CsI(Na) detectors (as shown in Figure 7) are given in Figure 8a,b, respectively. Apparent from each of these spectra is the notable disparity in the peak size derived by the single detector versus the triple detector systems. With three times the total active detection volume, the intensity of the peaks is observed to be approximately 3.3-times that of a single-unit platform.

To achieve the greatest sensitivity for any on-ground variability, the detector(s) were modelled (still applying the same single point source geometry as during earlier parts of the study) with their long-axes parallel to the surface of the ground. While such a sensor geometry results in an increased count-rate and a greater field-of-view of the detectors (ground sampling area/footprint), obtaining the greatest count-rate is viewed as more important than precisely defining the source(s) locality (e.g., through applying detector collimation) [6], as deconvolution and geometry optimisation algorithms are now increasingly capable and more accurate in interpolating an emitters exact position provided that sufficient gross count rates are obtained during an airborne survey. 

### 3.5. Implications for UAV Radiation Mapping

It, therefore, appears that small volume NaI and CsI-based detectors do not provide sufficient energy or spectral resolution for mineral survey applications as inherent broad peak widths detrimentally prohibit such detectors ability to identify other contributing radionuclides that may exist, and that an alternative is required. Candidates for this are lanthanide halides, such as LaBr_3_ and CeBr_3_—although these are not without their issues.

Firstly, these materials are relatively expensive by comparison, due to a combination of costs associated with materials and crystal growth. Secondly, while LaBr_3_ would be preferred, since its scintillator characteristics are marginally better than those of CeBr_3_ (leading to a better light yield and minimum detectable activity), a significant consideration is the inherent radioactivity of ^138^La, which has a decay peak at 1.436 MeV [43]. This emission would likely interfere with the detection of the ^40^K peak at 1.461 MeV, unless well-characterised and accounted for (the quantification of which is currently in-progress). Finally, although related to the previous two points, the ideal case would be to have isotopically pure lanthanum, in order to remove the interference from the ^128^La decay. However, in order to achieve this, further costs are associated with material processing. Alternatively, good calibration would be able to minimise the effects of this and permit the usage of this material.

## 4. Conclusions

The respective scintillation properties of CsI(Na), CsI(Tl), LaBr_3_ and LYSO(Ce) were modelled in GEANT4 with the aim of identifying which of these materials is best suited to environmental radiation monitoring purposes. LaBr_3_ was found to be the best of this selection, where its superior detection efficiency leads to increased count rates and luminosity peak heights, with excellent spectral resolution. It, therefore, represents one of the better choices for monitoring purposes when coupled with PMTs/SiPMs. However, due to its self-radioactivity, and the resultant increase in the signal to noise ratio at low energy, CeBr_3_ can be considered as a close competitor, though further investigation is required to definitively prove which of these materials is superior. More work is also required in order to find an appropriate way of shielding the crystals from atmospheric exposure so as to mitigate their hygroscopic behaviour.

## Figures and Tables

**Table 1:** Summary of key properties for candidate scintillator materials.

Material	Peak Emission (nm)	Light Yield (ph/MeV)	Density (g/cm^3^)	Attenuation @ 1.5 MeV	Energy Resolution (@ 661.7 keV)	Decay Time (ns)	References
NaI(Tl)	415	38,000–55,000	3.67	114.7	7%	250	[18,19]
CsI(Na)	420	38,000–44,000	4.51	140.9	5.8%	630	[18,20,21]
CsI(Tl)	540–550	52,000–65,000	4.51	140.9	6.9%	1000	[18,21,22,23]
BGO	480	8,000–10,000	7.13	222.8	9.7–16%	300	[18,24,25]
GAGG(Ce)	520	22,000–60,000	6.63	207.2	5.1%	87	[26,27,28,29,30]
LYSO(Ce)	420	30,000–33,000	7.1–7.2	225.0	8-20%	45	[25,31,32]
LaBr_3_	380	63,000	5.08–5.22	158.8	2.6–3.5%	16	[18,33,34,35]
CaF_2_(Eu)	435	19,000–30,000	3.19	99.7	5.4%	950	[18,36]
CeBr_3_	380–390	57,000–66,000	5.1–5.2	159.4	3.8–4%	18–20	[36,37,38]
SrI_2_(Eu)	435	80,000–115,000	4.55	142.2	2.8–4%	1200	[18,35,36,39,40]

## References

[1] Steinhauser G., Brandl A., Johnson T.E. (2014). Comparison of the Chernobyl and Fukushima nuclear accidents: A review of the environmental impacts. Sci. Total Environ..

[2] Hu Q.-H., Weng J.-Q., Wang J.-S. (2010). Sources of anthropogenic radionuclides in the environment: A review. J. Environ. Radioact..

[3] Lee W.E., Ojovan M.I., Jantzen C.M. (2013). Radioactive Waste Management and Contaminated Site Clean-Up: Processes, Technologies and International Experience.

[4] Tyler A.N., Sanderson D.C.W., Scott E.M., Allyson J.D. (1996). Accounting for spatial variability and fields of view in environmental gamma ray spectrometry. J. Environ. Radioact..

[5] Connor D., Martin P.G., Scott T.B. (2016). Airborne radiation mapping: Overview and application of current and future aerial systems. Int. J. Remote Sens..

[6] IAEA (1991). Airborne Gamma Ray Spectrometer Surveying.

[7] Minty B. (1997). Fundamentals of airborne gamma-ray spectrometry. AGSO J. Aust. Geol. Geophys..

[8] Schwarz G.F., Rybach L., Klingele E.E. (1995). Data Processing and Mapping in Airborne Radioactivity Surveys.

[9] Pitkin J.A., Duval J.S. (1980). Design parameters for aerial gamma-ray surveys. Geophysics.

[10] Towler J., Krawiec B., Kochersberger K. (2012). Radiation Mapping in Post-Disaster Environments Using an Autonomous Helicopter. Remote Sens..

[11] Sanada Y., Torii T. (2015). Aerial radiation monitoring around the Fukushima Dai-ichi Nuclear Power Plant using an unmanned helicopter. J. Environ. Radioact..

[12] Connor D.T., Martin P.G., Smith N.T., Payne L., Hutton C., Payton O.D., Yamashiki Y., Scott T.B. (2018). Application of airborne photogrammetry for the visualisation and assessment of contamination migration arising from a Fukushima waste storage facility. Environ. Pollut..

[13] Connor D.T., Martin P.G., Pullin H., Hallam K.R., Payton O.D., Yamashiki Y., Smith N.T., Scott T.B. (2018). Radiological comparison of a FDNPP waste storage site during and after construction. Environ. Pollut..

[14] Martin P.G., Payton O.D., Fardoulis J.S., Richards D.A., Scott T.B. (2015). The use of unmanned aerial systems for the mapping of legacy uranium mines. J. Environ. Radioact..

[15] MacFarlane J.W., Payton O.D., Keatley A.C., Scott G.P.T., Pullin H., Crane R.A., Smilion M., Popescu I., Curlea V., Scott T.B. (2014). Lightweight aerial vehicles for monitoring, assessment and mapping of radiation anomalies. J. Environ. Radioact..

[16] Martin P.G., Payton O.D., Fardoulis J.S., Richards D.A., Yamashiki Y., Scott T.B. (2016). Low altitude unmanned aerial vehicle for characterising remediation effectiveness following the FDNPP accident. J. Environ. Radioact..

[17] Salek O., Matolin M., Gryc L. (2018). Mapping of radiation anomalies using UAV mini-airborne gamma-ray spectrometry. J. Environ. Radioact..

[18] Knoll G.F. (2010). Radiation Detection and Measurement.

[19] Sodium Iodide (Tl) Scintillator Crystal. https://www.advatech-uk.co.uk/nai_tl.html.

[20] CsI(Na)Crystal, CsI(Na) Scintillation Crystal, CsI Crystal; Kinheng Crystal Material (Shanghai)Co., Ltd. http://www.kinheng-crystal.com/scintillation-crystal/csina-scintillation-crystal-csina-scintillator.html.

[21] CsI(Tl), CsI(Na) Cesium Iodide Scintillation Material. www.crystals.saint-gobain.com/sites/imdf.crystals.com/files/documents/csitl-and-na-material-data-sheet.pdf.

[22] Hilger Crystals—Crystal Material Datasheet Thallium doped Caesium Iodide CsI. www.dynasil.com//assets/CsITl.pdf.

[23] CsI(Tl)Crystal, CsI(Tl) Scintillation Crystal, CsI Crystal; Kinheng Crystal Material (Shanghai)Co., Ltd. http://www.kinheng-crystal.com/scintillation-crystal/csi-tl-scintillator-csi-tl-scintillation-crystal.html.

[24] Bismuth Germanate Scintillation Material. www.crystals.saint-gobain.com/sites/imdf.crystals.com/files/documents/bgo-material-data-sheet.pdf.

[25] BGO and LYSO Crystals; Omega Piezo. http://www.omegapiezo.com/crystal-scintillators/.

[26] Seitz B., Campos Rivera N., Stewart A.G. (2016). Energy Resolution and Temperature Dependence of Ce:GAGG Coupled to Silicon Photomultipliers. IEEE Trans. Nucl. Sci..

[27] Kim H.L., Kim H.J., Jang E.J., Lee W.G., Ki M.K., Kim H.D., Jun G.S., Kochurikhin V. (2015). Scintillation properties of the Gd_3_Al_2_Ga_3_O_12_:Ce crystal. J. Ceram. Process. Res..

[28] Rawat S., Tyagi M., Netrakanti P.K., Kashyap V.K.S., Mitra A., Singh A.K., Desai D.G., Kumar G.A., Gadkari S.C. (2016). Pulse shape discrimination properties of Gd_3_Ga_3_Al_2_O_12_:Ce, B single crystal in comparison with CsI:Tl. Nucl. Instruments Methods Phys. Res. Sect. A Accel. Spectrometers Detect. Assoc. Equip..

[29] Kamada K., Shimazoe K., Ito S., Yoshino M., Endo T., Tsutsumi K., Kataoka J., Kurosawa S., Yokota Y., Takahashi H. (2014). Development of a Prototype Detector Using APD-Arrays Coupled With Pixelized Ce:GAGG Scintillator for High Resolution Radiation Imaging. IEEE Trans. Nucl. Sci..

[30] Sibczynski P., Iwanowska J., Moszyński M., Swiderski L., Szawlowski M., Kamada K., Yoshikawa A., Sato H. (2013). Characterization of new GAGG:Ce scintillators with different Al-to-Ga ratio. Proceedings of the 2013 IEEE Nuclear Science Symposium and Medical Imaging Conference (2013 NSS/MIC).

[31] PreLude^TM^ 420 crystal Cerium doped Lutetium|Products|Saint-Gobain Crystals. https://www.crystals.saint-gobain.com/products/prelude-420-LYSO.

[32] LYSO:Ce Crystal—LYSO(Ce) Scintillator; Epic Crystal Co., Ltd. http://www.epic-crystal.com/lysoce/lyso(ce)-scintillator.html.

[33] BrilLanCe^TM^ 380 crystal Lanthinum Bromide LaBr_3_(Ce)|Products|Saint-Gobain Crystals. https://www.crystals.saint-gobain.com/products/standard-and-enhanced-lanthanum-bromide.

[34] Latest Lanthanum Bromide(LaBr_3_:Ce) crystal experiment results. http://www.epic-crystal.com/feature-design/latest-lanthanum-bromide.html.

[35] Cherepy N.J., Payne S.A., Asztalos S.J., Hull G., Kuntz J.D., Niedermayr T., Pimputkar S., Roberts J.J., Sanner R.D., Tillotson T.M. (2009). Scintillators with Potential to Supersede Lanthanum Bromide. IEEE Trans. Nucl. Sci..

[36] Radiation Detection Materials. http://www.hellma-materials.com/text/989/en/ausblenden/hellma-materials-scintillation-crystals.html.

[37] Cerium Bromide (CeBr_3_) Scintillators | Berkeley Nucleonics. https://www.berkeleynucleonics.com/cerium-bromide.

[38] Quarati F.G.A., Dorenbos P., Van Der Biezen J., Owens A., Selle M., Parthier L., Schotanus P. (2013). Scintillation and detection characteristics of high-sensitivity CeBr_3_ gamma-ray spectrometers. Nucl. Instruments Methods Phys. Res. Sect. A Accel. Spectrometers Detect. Assoc. Equip..

[39] Strontium Iodide (Eu)—SrI2(Eu) Scintillator Crystal. https://www.advatech-uk.co.uk/sri2_eu.html.

[40] CapeSym|ScintiClear^TM^. http://www.capesym.com/sri2.html.

[41] Birks J.B. (1964). Theory and Practice of Scintillation Counting.

[42] Agostinelli S., Allison J., Amako K., Apostolakis J., Araujo H., Arce P., Asai M., Axen D., Banerjee S., Barrand G. (2003). GEANT4—A simulation toolkit. Nucl. Instruments Methods Phys. Res. Sect. A Accel. Spectrometers Detect. Assoc. Equip..

[43] Menge P.R., Gautier G., Iltis A., Rozsa C., Solovyev V. (2007). Performance of large lanthanum bromide scintillators. Nucl. Instruments Methods Phys. Res. Sect. A Accel. Spectrometers Detect. Assoc. Equip..

[44] Guss P., Reed M., Yuan D., Reed A., Mukhopadhyay S. (2009). CeBr3 as a room-temperature, high-resolution gamma-ray detector. Nucl. Instruments Methods Phys. Res. Sect. A Accel. Spectrometers Detect. Assoc. Equip..

[45] Mioduski T., Gumiński C., Zeng D., Voigt H. (2013). IUPAC-NIST Solubility Data Series. 94. Rare Earth Metal Iodides and Bromides in Water and Aqueous Systems. Part 2. Bromides. J. Phys. Chem. Ref. Data.

[46] Iltis A., Mayhugh M.R., Menge P., Rozsa C.M., Selles O., Solovyev V. (2006). Lanthanum halide scintillators: Properties and applications. Nucl. Instruments Methods Phys. Res. Sect. A Accel. Spectrometers Detect. Assoc. Equip..

